# Effect of Continuous Mixer Design and Parameters on the Degradation of Polylactic Acid

**DOI:** 10.3390/polym17111568

**Published:** 2025-06-04

**Authors:** Mansour Alotaibi, Jainam Shah, Aniket Sadani, Carol Forance Barry

**Affiliations:** Department of Plastics Engineering, University of Massachusetts Lowell, Lowell, MA 01854, USAjainam_shah@student.uml.edu (J.S.);

**Keywords:** polylactic acid, process-induced degradation, continuous mixer

## Abstract

Polylactic acid (PLA) has gained attention as a sustainable, compostable polyester, but process-induced degradation in single- and twin-screw extruders reduces PLA’s molecular weight and affects its properties. In addition, PLA is often blended with other materials to improve its properties. A continuous mixer, which provides tighter control of shear levels and lower processing temperatures, produces less degradation of heat-sensitive polymers like polyvinyl chloride, but there is limited information about the effects of machine design and processing parameters. Therefore, this work investigated three parameters in the mixer section (rotor design, rotor speed, and orifice position) and screw speeds in the extruder section when processing PLA using a continuous mixer. The resultant PLA samples were characterized for their rheological, thermal, and chemical structure properties. It was found that higher rotor speeds and smaller orifice openings resulted in lower molecular weights, whereas varying the screw speed in the extruder did not significantly affect the molecular weight. Rotor design substantially impacted degradation, with rotors that provided lower shear stress and residence time producing very low reductions in molecular weight. Overall, this work provided insight on how to select rotors and processing parameters to reduce degradation of PLA for continuous mixer

## 1. Introduction

The global demand for plastic materials is expected to continue to increase due to their excellent mechanical properties, light weight, chemical resistance, and low cost [1,2,3,4]. The resultant increased global production of plastic materials will present several environmental challenges. Landfilling will produce significant increases in non-degradable plastics of all types if this waste is not recycled [5]. Air pollution and carbon dioxide emissions from the incineration of degradable plastic waste will increase [6]. Additionally, there will be dependence on non-renewable resources, i.e., fossil fuels, to produce conventional plastic materials such as polyethylene, polypropylene, and polystyrene [7]. Using biobased and biodegradable polymers is a promising solution to these challenges, as they decrease dependence on fossil fuels and, with proper composting systems, can reduce the accumulation of plastic waste [8,9].

**Figure 1:**
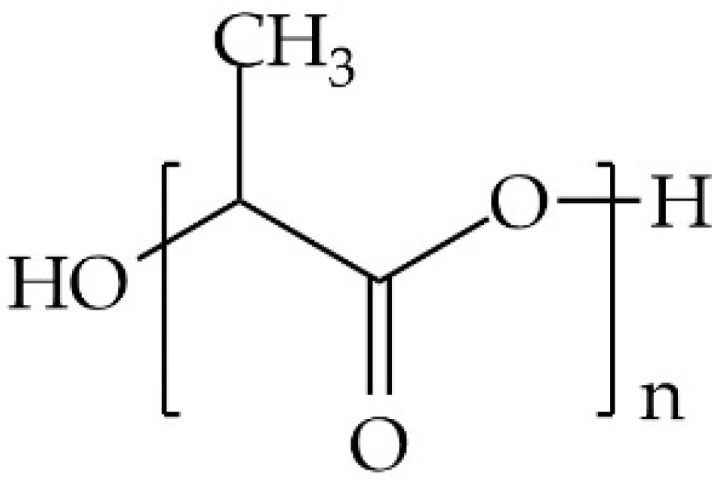
Chemical structure of PLA.

Among the sustainable alternatives to non-degradable polymeric materials, polylactic acid (PLA) is a biobased aliphatic polyester (Figure 1) that is derived from renewable resources such as corn and sugar cane [6,10,11]. PLA can be degraded in industrial composting facilities [12], can be melt processed using techniques such as extrusion and injection molding, and is commercially available at large scale [13,14]. The ester group in PLA’s backbone that is responsible for biodegradation, however, is a susceptible linkage during melt processing [14,15]. Chain scission at the ester groups results in significant reductions in PLA’s molecular weight after melt forming and melt mixing [7]. Melt mixing is an essential step after polymer production to produce a uniform pelletized product and incorporate additives such as antioxidants. Moreover, customized and specialty products can be manufactured by melt mixing multiple components to create polymer blends and polymers with a range of additives.

When melt mixing PLA compounds, higher melt temperatures [1,5,16,17,18,19,20,21,22] and high moisture contents [16,17,18,19] are the most significant factors affecting degradation [1,5,16,18,19,20,21,22,23]. The recommended moisture content is below 250 ppm [24]. The presence of moisture during melt processing accelerates the hydrolytic degradation of PLA, leading to a reduction in molecular weight [16,17,18,19]. In contrast, a processing temperature greater than 200 °C increases the chain scission reactions, as reported by Mysiukiewicz et al. [20] for four PLA grades extruded at temperatures between 180 °C and 260 °C in a co-rotating twin-screw extruder. Moreover, higher shear stresses [25] and prolonged extruder residence times [18] increase degradation. The resultant thermal, thermomechanical, and hydrolytic degradation affects PLA’s processability and final product properties. In addition, more effective thermal stabilization, chain extender additives, and processing in the presence of a nitrogen atmosphere can prevent PLA from degrading and improve its properties [16,22].

Co-rotating twin-screw extruders commonly are used for melt compounding of PLA [26]. With these extruders, the screw speed and screw configuration can induce shear stresses, leading to reductions in melt viscosity due to chain scission. Although higher screw speeds generally reduce residence time, they impart more shear and increase degradation of PLA [25]. In addition, the screw configuration (program) can significantly impact the level of shear stresses applied to the material and the residence time in the extruder. A screw configuration with more kneading blocks can increase the intensity of mixing and shearing as well as the extruder residence time. For example, Kosmalska et al. [5] reported that a screw configuration with four kneading and mixing zones produced a higher reduction in the molecular weight of PLA compared to a screw design with two kneading zones. Overall, the degradation of PLA during melt mixing can be reduced by lowering the processing temperatures, reducing the residence time, and using less aggressive screw designs.

The extruder type directly influences the degradation of PLA by influencing the residence time and shear stresses that are applied during processing. The process-induced degradation of PLA has been extensively investigated using co-rotating twin-screw extruders [5,17,20,21], counter-rotating twin-screw extruders, and single-screw extruders [1,22,27,28,29]. Scaffaro et al. [30] found that processing PLA using a co-rotating twin-screw extruder, a counter-rotating twin-screw extruder, and a single-screw extruder decreased the PLA’s molecular weight by 7%, 36%, and 37%, respectively. Compared to the co-rotating twin-screw extruder, the counter-rotating twin-screw extruder increased the residence time, whereas the single-screw extruder provided greater shear stress. With a co-rotating quad-screw extruder, the longer residence time and greater shear stresses applied by the three intermeshing zones of the four parallel screws produced a 9% reduction in PLA’s molecular weight compared to a 7% reduction with a co-rotating twin-screw extruder having the same diameter, length-to-diameter ratio, and screw design [25].

**Figure 2:**
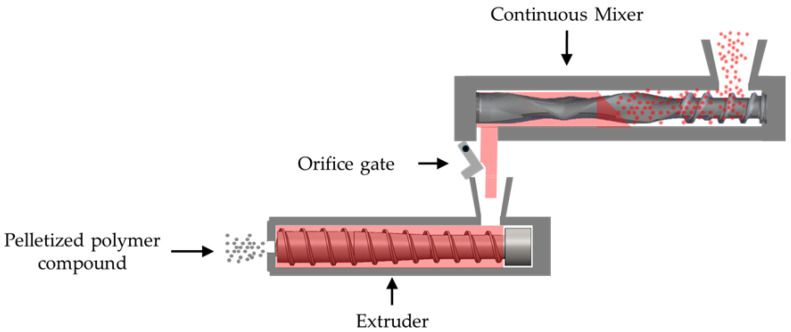
Schematic showing a continuous mixer in which resin pellets (red dots) flowed from the feeder, through the continuous mixer, through the orifice gate, and through the single-screw extruder; the compounded polymer exited the strand die and was pelletized (gray dots).

Other mixing equipment can be used for melt compounding PLA. As illustrated in Figure 2, a continuous mixer is a two-stage compounding system consisting of a mixing unit with two counter-rotating, non-intermeshing rotors followed by a single-screw extruder or gear pump. These continuous mixers employ lower processing temperatures, lower mechanical shear, and lower specific energies [31]. The rotor design, however, directly impacts the shear forces within the mixer section. Mixing of highly filled compounds of heat-stable polymers generally is performed with one rotor design (style 15), whereas a low-shear rotor design (style 7) typically is used for heat-sensitive polyvinyl chloride materials. Recent work with high-density polyethylene (HDPE) and nine rotor designs showed that the rotor design influences the melt temperature rise in the mixer [32]; that increase in melt temperature can contribute to degradation of PLA. The performance of continuous mixers has been compared with the performance of other melt compounding systems, primarily using heat-stable materials [33,34,35,36,37]. Lahmann and Knowlton [34], however, evaluated the molecular weight retention of PLA with different levels of talc filler using a laboratory-scale continuous mixer with a mixed-style 7 and 15 rotor design and a co-rotating twin-screw extruder at fixed processing conditions. They reported a 1% reduction in the molecular weight of the unfilled PLA when using a continuous mixer compared to a 7% reduction for a co-rotating twin-screw extruder; this performance was attributed to the lower set temperatures and lower shear levels in the continuous mixer.

Studies on the processing of PLA using continuous mixers, however, remain limited, with the one reported study employing a fixed rotor design and specific processing parameters [34]. This limited work results in a lack of understanding of the impacts of rotor design and processing parameters on the PLA degradation in continuous mixers. Therefore, the objective of this work was to evaluate the impact of rotor design, rotor speed, orifice position, and speed of the single screw on the process-induced degradation of PLA using a continuous mixer. The degradation of the PLA was assessed by melt flow index, rheological properties, which provided molecular weight, and changes in chemical structure and thermal properties. Overall, this work provided insight into how the selection of rotors and processing parameters can reduce degradation of PLA for continuous mixers.

## 2. Materials and Methods

### 2.1. Materials

A commercial grade of polylactic acid (PLA) with a melt flow rate of 6 g/10 min (210 °C/2.16 kg) supplied by NatureWorks LLC (Ingeo™ Biopolymer 2003D, Minnetonka, WI, USA). To prevent hydrolysis, the PLA was dried using a desiccant dryer (Dri-Air Industries, Inc., model: MPD-30D, East Windsor, CT, USA) at 40 °C for 12 h prior to processing and characterization.

### 2.2. Continuous Mixer Trials

The PLA processing was performed using a Farrel Pomini Laboratory Compact Processor (model: CPeX^®^, Ansonia, CT, USA). This melt compounding equipment included a mixing chamber with an adjustable gate orifice and a single-screw extruder positioned downstream. The mixing section featured a 35 mm diameter barrel and 33 mm diameter rotors. The hot-fed extruder had a 60 mm diameter single screw with a length-to-diameter ratio (L/D) of 11:1 and a compression ratio of 2.3:1. The PLA pellets were starve-fed to the mixing chamber using a volumetric feeder (Brabender Technologie, model FV18-0 V0L, Mississauga, ON, Canada). The melt exited the mixer’s extruder through a triple-strand die; the extruded strands were cooled and solidified in a water bath and then pelletized using a strand pelletizer (Bay Plastics Machinery Company LLC, Bay City, MI, USA).

**Figure 3:**
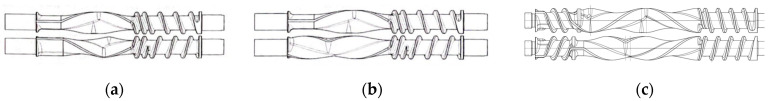
Rotor designs used in experimental trials: (**a**) style 15 (15L/15R std) and (**b**) combinations of styles 7 and 15 (15L/7R std), and (**c**) HD rotor with combinations of styles 7 and 15 (15L/7R HD).

The rotor designs employed in this study are illustrated in Figure 3. The rotor designs included a combination of styles 7 and 15 (15/7 std) and a new high dispersion (HD) rotor (15/7 HD). Additionally, style 15 (15/15) was used as the reference design, as it is the most widely used rotor design. These rotor designs were selected based on their mean residence time and the rise in melt temperatures for HDPE [32]. The rotor designs that produced the highest and lowest residence time and rise in mixer melt temperature were the styles 15/7 HD and 15/7 std, respectively. The 15/7 std and 15/15 std rotors had two sections: one for conveying and one for melting and mixing. The overall length of these rotors was 205 mm, and the mixing section length was 122 mm. The HD rotor, however, was 328 mm in length and featured a 201 mm long mixing section bracketed by two conveying sections. Melting in the high dispersion (HD) rotors occurs further up-stream than with the standard rotors.

Table 1 provides an overview of the experimental design for evaluation of the effects of mixer variables on the degradation of PLA. The one-factor-at-a-time (OFAT) design of experiments (DOE) incorporated three rotor designs and three levels of rotor speed and orifice opening. The rotor speeds of 400, 600, and 800 rpm and the orifice openings of 10, 30, and 50% were selected based on the results from a prior full factorial study [32]. The feed rate was held constant at 20 kg/h and the extruder speed was fixed at 40 rpm. Although feed rate affects the fill level in the mixer, prior work [32] showed that it had less significant impact compared to rotor speed and orifice opening. The set temperatures for the second zone of the mixer chamber, the orifice, the extruder, and the die were held constant at 180 °C, which is the lowest processing temperature recommended for PLA [24] (Table 2). The set temperature for the first zone of the mixer chamber was fixed at 80 °C for the standard rotors and 120 °C for the high dispersion (HD) rotors, where melting started earlier than with the standard rotors; these lower temperatures prevented premature melting of PLA. For all experimental trials, the continuous mixer was run with the selected parameters for 20 min prior to data collection to ensure steady-state conditions. The melt temperatures were recorded from the machine’s readouts for the mixer chamber and single-screw extruder; the thermocouples were located at the discharge end of the mixer and the extruder. When the mixing process reached a steady state condition, the samples of extrudate were collected. During each 10 min long trial, the melt temperatures remained constant; these temperatures were reported as the melt temperatures for the mixer and the extruder. In addition, a separate hand-held thermocouple (Omega Engineering Inc., Norwalk, CT, USA) was used to confirm the melt temperatures for both the mixing section and the extruder; this measurement time was about 1 min.

The Farrel continuous mixer is a two-stage process in which the mixer section and extruder section operate independently. Since it is crucial to understand the contribution of the extruder section to the degradation of PLA, the screw speed for the extruder section was investigated. As shown in Table 3, the investigation of the extruder section was conducted using the 15/7 std rotor because it produced the lowest rise in mixer melt temperature. The two extruder screw speeds were 40 and 80 rpm, whereas the rotor speeds were 400, 600, and 800 rpm, and the orifice openings were 10%, 30%, and 50%. All other parameters were the same as for the evaluation of the mixing section.

### 2.3. Characterization

#### 2.3.1. Rheological Properties

The rheological properties of the processed PLA samples were determined using an extrusion plastometer (Dynisco, model: 710-1-5-010-14, Franklin, MA, USA) and a parallel plate rheometer (TA Instruments, model: ARES-G2, New Castle, DE, USA). The melt flow index (MFI) measurements were conducted using an extrusion plastometer and ASTM D1238-20 (Procedure A); the set temperature was 210 °C, and the weight load was 2.16 kg. For each sample, three measurements were carried out, and the average value was calculated. For the parallel plate rheometer, specimen disks with a diameter of 25 mm and a thickness of 1.5 mm were prepared using a micro-injection molding machine (Xplore Instruments BV, model M-12, Sittard, The Netherlands) at a temperature of 180 °C and a soak time of 3 min. For rheological measurements, the set temperature was 180 °C. A strain sweep was first performed over a strain range of 0.1% to 100% at a fixed frequency of 10 Hz to determine the range of linear viscoelastic behavior of the PLA. Then, frequency sweep measurements were performed over a frequency range of 0.02 to 15.92 Hz at a constant strain of 2%. For the resultant complex viscosity-frequency data, Cox–Merz rules were applied to obtain the corresponding shear viscosities and shear rates. These data were fitted to the Carreau–Yasuda model [38] using Trios 5.0 software (TA Instruments, New Castle, DE, USA) to determine the zero-shear viscosities.

The molecular weight (Mw) was estimated from zero-shear viscosities (η_0_) of the processed and unprocessed PLA by using [39]:

(1) $$\eta_{0}={k(M_{w})}^{a}$$

where a was 3.4, and k was 5.5 × 10^−15^.

#### 2.3.2. Fourier Transform Infrared Spectroscopy

**Figure 4:**
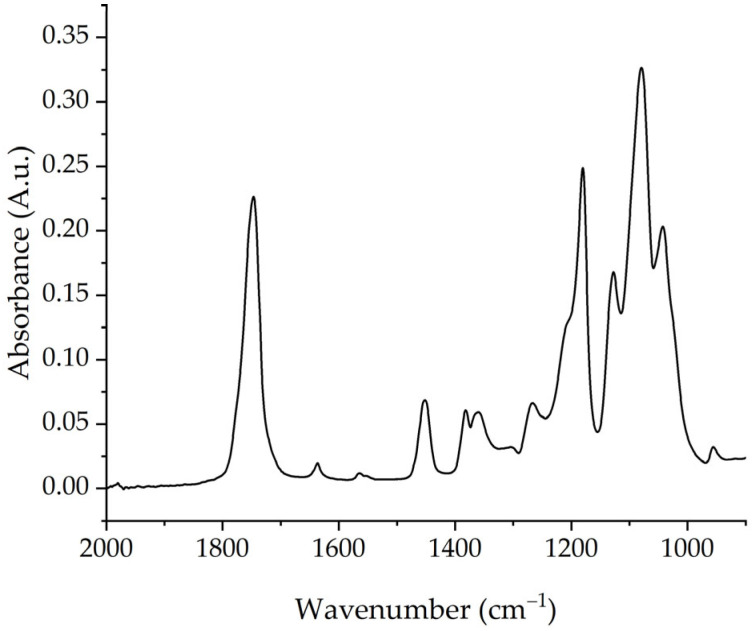
Unnormalized Fourier-transform infrared spectroscopy (FTIR) spectra for the unprocessed PLA.

Fourier-transform infrared (FTIR) spectra of the processed PLA samples were obtained using an FTIR spectrometer (Nicolet™ iS50, Thermo-Fisher Scientific, Waltham, MA, USA) in ATR mode. The test specimens were injection-molded 25 mm diameter disks with a thickness of 1.5 mm. The measurements were performed at 4 cm^−1^ resolution, 64 scans, and a wavenumber range of 400–4000 cm^−1^ in the absorbance mode. Figure 4 shows the spectra for the unprocessed PLA. The resultant spectra were normalized using the absorbance value at 1455 cm^−1^, which corresponds to the asymmetric bending of the CH_3_ group; this value is recognized as a suitable internal standard for PLA [22,39,40,41]. To ensure accuracy, at least two replicates of each sample were performed, and the average results are presented with standard deviations.

#### 2.3.3. Differential Scanning Calorimetry

The thermal properties of the unprocessed and processed PLA were characterized using a differential scanning calorimeter (Mettler Toledo, DSC 3+, Columbus, OH, USA). The differential scanning calorimetry (DSC) measurements were performed in a nitrogen atmosphere; the samples (5–10 mg) underwent two heating cycles and one cooling cycle from 25 °C to 200 °C at a rate of 10 °C/min. To ensure accurate results, at least two replicates were conducted for each sample. The data from the second heating cycle were analyzed using STARe software (Mettler Toledo, Columbus, OH, USA) to determine the cold crystallization temperature, melt temperature, and percent crystallinity of the unprocessed and processed PLA samples. Percent crystallinity of the samples was based on the melting enthalpy of 100% crystalline PLA (93 J/g) [42].

## 3. Results and Discussion

### 3.1. Processing Parameters

**Figure 5:**
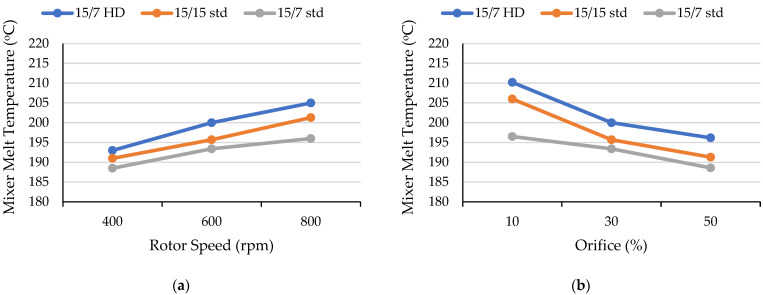
Effect of (**a**) rotor speed with a fixed 30% orifice opening and (**b**) orifice position when the screw speed was 600 rpm on the mixer melt temperature obtained with three rotor designs.

Melt temperature is a critical processing parameter when processing heat-sensitive materials such as PLA. A significant rise in melt temperature due to viscous dissipation and longer residence times can result in thermomechanical degradation. Thus, monitoring melt temperature changes is a useful approach to optimizing processing parameters. As shown in Figure 5a, mixer melt temperature increased nearly linearly when the rotor speed increased from 400 rpm to 800 rpm (since the stabilized mixer conditions provided constant melt temperatures, there were no error bars). Increasing the rotor speed led to higher shear forces, resulting in greater viscous dissipation and elevated mixer melt temperatures. This trend was consistent with previously reported findings for continuous mixers [32,33]. The mixer’s melt temperature increased 10 °C for the 15/15 std rotor, 7 °C for the 15/7 std rotor, and 12 °C for the 15/7 HD rotor (compared to the 400 rpm rotor speed). The 15/7 HD rotor consistently generated the highest melt temperatures (193 °C to 205 °C) because its longer (201 mm) mixing section produced a longer residence time of 90 s. In contrast, the style 15/7 std rotors exhibited the lowest temperatures (189 °C to 196 °C) since the combination of the style 15 and style 7 rotors created a longer apex zone (i.e., 25 mm compared to 20 mm for the style 15/15 rotors) and a neutral section on only one rotor; this design provided an intermediate shear stress and the lowest residence time. The 15/15 std rotor produced an intermediate rise in temperature (191 °C to 201 °C), which resulted from the greater shear and residence time associated with its two 35 mm long neutral sections.

**Figure 6:**
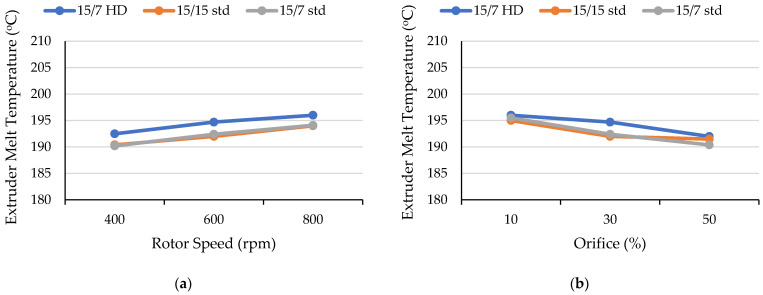
Effect of (**a**) rotor speed with a fixed 30% orifice opening and (**b**) orifice position when the screw speed was 600 rpm on the extruder melt temperature obtained with three rotor designs.

On the other hand, the mixer melt temperature decreased significantly with larger orifice openings (Figure 5b). As the orifice opening increased from 10% to 50%, the mixer temperature decreased from 206 °C to 191 °C for the 15/15 std rotors, 197 °C to 189 °C for the 15/7 std rotors, and from 210 °C to 196 °C for the 15/7 HD rotors. This behavior mainly was attributed to the decreased backflow with larger orifice openings. Although the residence times actually were lower with smaller orifice openings, greater shear heating due to increased backflow reduced the viscosity of the PLA melt. As a result of backflow, the mixer torque increased as the orifice opening decreased. The torque increased from 30% to 59% for the 15/7 std rotor design, 50% to 61% for the 15/15 std rotor design, and 60% to 66% for the 15/7 HD rotor design. As shown in Figure 6, varying the rotor speed and orifice position produced comparable extruder melt temperatures for all rotor designs. The 15/7 HD rotor provided a slight increase in overall extruder melt temperature, but the 15/15 std and 15/7 std rotors exhibited the same melt temperatures. This behavior was attributed to the single-extruder function in the Farrel continuous mixer, which pumps the polymer melt that has already been melted in the mixer section [32,34].

**Figure 7:**
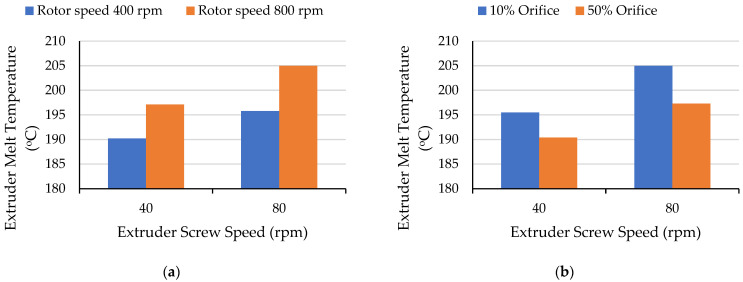
Effect of extruder screw speed on the extruder melt temperature with (**a**) a variable rotor speed and a constant orifice of 30% and (**b**) variable orifice and constant rotor speed of 600 rpm.

Figure 7a illustrates the effect of extruder screw speed on extruder melt temperature. With a fixed orifice opening of 30% and a rotor speed of 400 rpm, the temperature was 190 °C at 40 rpm and increased by 6 °C when the screw speed was increased to 80 rpm. For a rotor speed of 800 rpm, the corresponding temperatures were about 197 °C and 11 °C. The orifice opening significantly affected the extruder melt temperature at different screw speeds (Figure 7b). When the orifice opening was 50%, the extruder melt temperature was about 195 °C at 40 rpm and increased by 7 °C as the screw speed was increased to 80 rpm. With a more restrictive orifice (10%), the extruder melt temperature was about 190 °C at 40 rpm and increased significantly (by 10 °C) with increasing screw speed. Higher extruder screw speeds generally increase shear heating. The greater rise in melt temperature with the faster rotor speed—which produced a higher mixer melt temperature and a shorter residence time—was probably due to greater backflow from the lower viscosity melt. Overall, both the screw speed and mixing section parameters impacted the extruder melt temperature.

### 3.2. Rheological Properties

**Figure 8:**
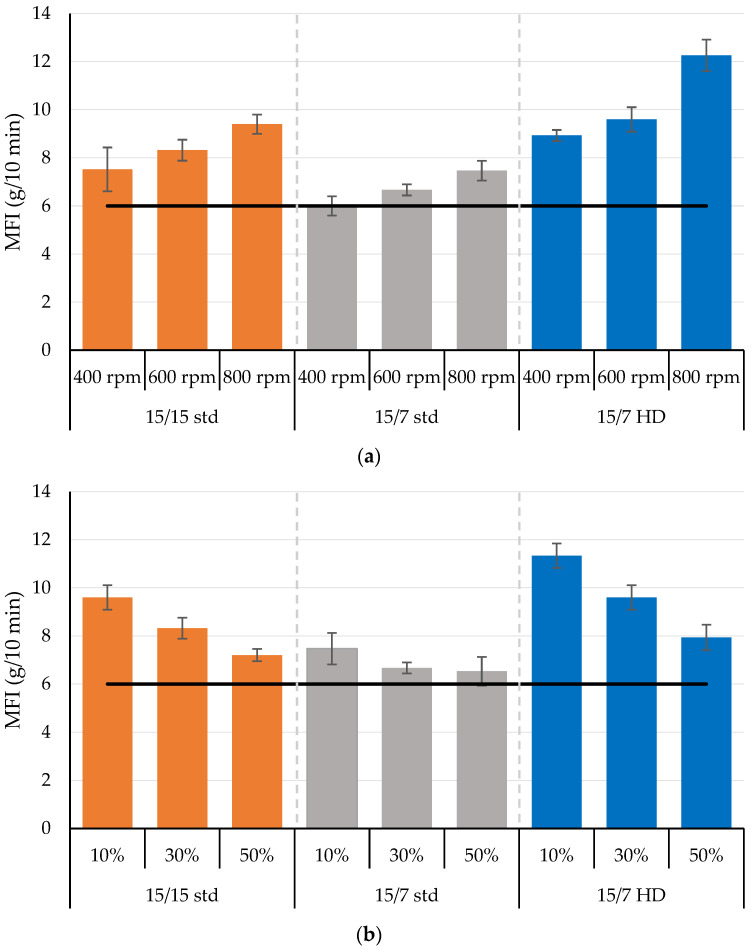
The dependence of the melt flow index of the processed PLA on (**a**) rotor speed at a fixed 30% orifice opening and (**b**) orifice position with a constant the screw speed of 600 rpm.

The melt flow index (MFI) is essential for understanding a material’s flowability, as it indicates changes in the polymer’s molecular weight and viscosity. Figure 8a illustrates the effect of rotor speed on the MFI of the processed PLA using different rotor designs. The MFI increased significantly with higher rotor speeds, indicating reductions in melt viscosity. Higher rotor speeds generate greater shear, leading to increased viscous dissipation, which reduces melt viscosity and can induce chain scission reactions in the processed PLA. These trends were consistent with increases in screw speed when processing PLA using twin-screw extruders [5,20]. For 15/15 std, 15/7 std., and 15/7 HD, the melt index increased linearly with increasing rotor speed at rates of 0.0047, 0.0038, and 0.0084 g/10 min per rpm, respectively; the corresponding coefficients of determination (*r*^2^) were 0.99, 0.99, and 0.89.

Rotor design also affected melt index. At a rotor speed of 400 rpm, the melt index increased by about 25%, 0%, and 49% compared to the melt index of the unprocessed PLA (6 g/10 min) for the 15/15 std rotors, 15/7 std rotors, and the 15/7 HD rotors, respectively. As the rotor speed increased from 400 rpm to 800 rpm, the corresponding increases in melt index were about 25%, 24%, and 37%. The trends for the rotors exhibited nearly linear relationships with the lengths of the rotor’s mixing sections; the relative increase in melt index at 400 and 600 rpm was 0.037 g/10 min per mm of mixing section length (*r*^2^ = 0.99), whereas this increase was 0.061 g/10 min per mm (*r*^2^ = 1.00). Since polymer was fully melted at the apex of the rotor’s mixing section, the mixing section length affects the residence time and backflow in the mixing section. The 15/7 std rotor has a 122 mm long mixing section, and the style 7 rotor does not have a neutral section at the end of the rotor; as a result, the mixing section’s length is about 122 mm. The 15/15 std rotor combines the 122 mm long mixing section with a 35 mm long neutral section to produce a mixing section length of 157 mm, whereas the 15/7 HD rotor has a mixing length of 201 mm and no neutral sections at the end of the rotors. Consequently, the 15/7 HD rotor exhibited greater backflow, viscous dissipation, and longer residence times in the longer mixing sections, which consistently increased the melt flow index. This increase was consistent with the increases in mixer melt temperatures, which would have reduced melt viscosity and induced chain scission in the PLA.

As shown in Figure 8b, the orifice position also affected the melt flow index of the PLA processed using different rotor designs. When the orifice opening was 10%, the melt flow indices increased by 60%, 25%, and 88% compared to the melt flow index of the unprocessed PLA (6 g/10 min) for the 15/15 std rotors, 15/7 std rotors, and the 15/7 HD rotors, respectively. Increasing the orifice opening from 10% to 50% decreased the corresponding relative melt flow index by 25%, 13%, and 29%. Larger orifice openings did not reduce mixer residence time. They, however, provided less flow restriction at the orifice, thereby reducing backflow inside the mixer section and producing less viscous dissipation. The increases in melt flow index with the 10% orifice opening and the reductions in relative melt flow index as the orifice was opened generally scaled with the length of the rotors’ mixing sections. This behavior suggests that smaller orifices combined with longer mixing sections produce significant reductions in melt viscosity and could provide greater process-induced degradation. With the rotors having the shortest mixing section (15/7 std), however, opening the orifice from 10% to 50% produced a much smaller improvement in melt flow index compared to the changes that occurred with the other two rotor designs (15/15 std and 15/7 HD). As reported in prior work [32], the mixer and total residence time increased with larger orifice openings. The mixer residence increased from 10.8 s to 12.5 s, 11.6 s to 15.2 s, and 19.4 s to 20.1 s for the 15/7 std, 15/15 std, and 7/15 HD rotors, respectively. The corresponding total residence times increased from 61.5 s to 64.5 s, 66.5 s to 70 s, and 70.6 s to 77.2 s. An ANOVA analysis showed that rotor design had the most significant impact on melt index compared to orifice position and rotor speed; the respective *p*-values were <0.0001, 0.0002, and 0.0003.

**Figure 9:**
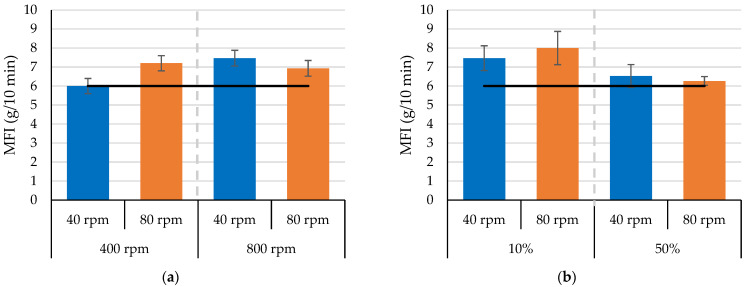
Melt flow index for the processed PLA as a function of screw speed at different (**a**) 15/7 std rotor speeds and a fixed orifice position of 30% and (**b**) orifice position with a fixed 15/7 std rotor speed of 600 rpm.

Figure 9 presents the effect of the continuous mixer’s extruder section on the melt flow index (MFI) of the processed PLA. Screw speed was examined at two different rotor speeds and two different orifice positions. The results reveal different behaviors when increasing the screw speed from 40 rpm to 80 rpm at different rotor speeds (at a fixed orifice of 30%) and orifice positions (at a fixed rotor speed of 600 rpm). As shown in Figure 9a, at a lower rotor speed (400 rpm), increasing the screw speed increased the average melt flow index of the processed PLA from 6 to 7.2 g/10 min. Similarly, as shown in Figure 9b, with a smaller orifice opening (10%), increasing the screw speed marginally increased the average MFI from 7.5 to 8 g/min. These results suggest that the combination of a lower rotor speed and a smaller orifice opening has a more pronounced effect on PLA degradation, resulting in a higher MFI, which indicates a reduction in PLA viscosity after processing. The lower rotor speeds increase residence time in the mixer chamber, while the smaller orifice opening restricts flow out of the mixer chamber.

Increasing the screw speed at a higher rotor speed (800 rpm), however, produced a marginal decrease in the MFI of the processed PLA (Figure 9a). The MFI decreased from 7.5 g/10 min to 6.9 g/10 min when the screw speed increased from 40 rpm to 80 rpm. Similarly, with a larger orifice opening (50%), the MFI decreased from 6.7 g/10 min to 6.2 g/10 min with an increase in screw speed from 40 rpm to 80 rpm (Figure 9b). The overall impact of screw speed on MFI was minimal compared to rotor speed and orifice positions. These results also suggest that the screw speed could help to minimize the PLA degradation; however, it varies according to the mixer section conditions (i.e., rotor speed and orifice position). Further study could provide a greater understanding of the interaction of the mixing section and the extruder section on the degradation of PLA.

**Figure 10:**
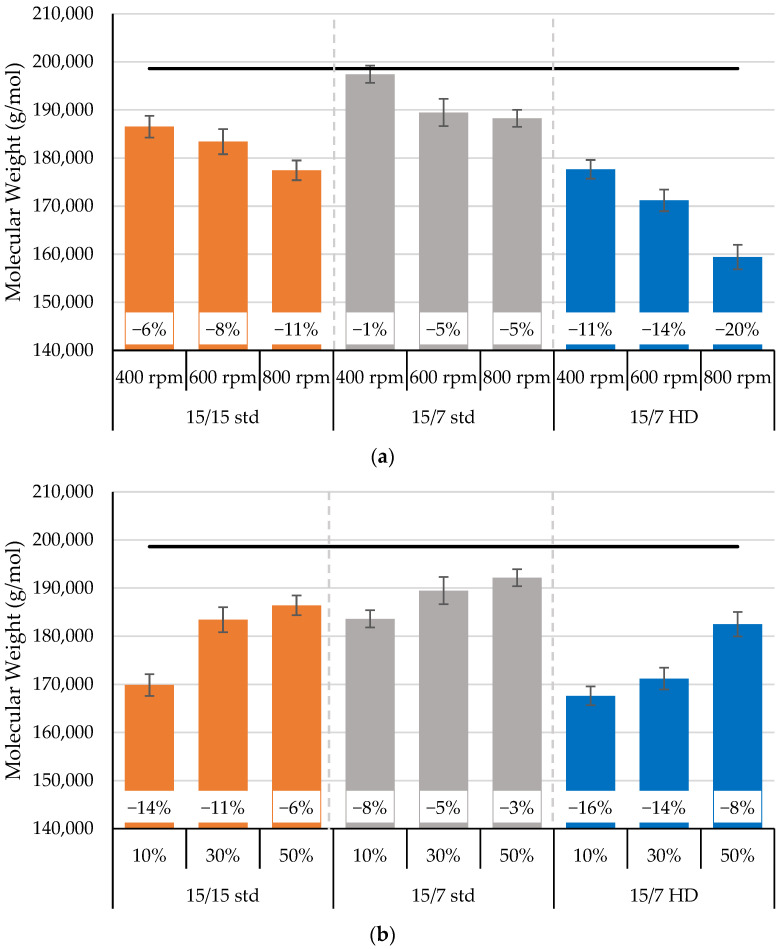
The dependencies of the reduction in the processed PLA’s molecular weight on (**a**) rotor speed with a fixed 30% orifice opening and (**b**) orifice position for a constant rotor speed of 600 rpm.

Since the melt flow index data indicated changes in molecular weight, the parallel-plate rheometer was used to determine the zero-shear viscosity. The molecular weights calculated from the zero-shear viscosities for the processed and unprocessed PLA are presented in Figure 10; the zero-shear viscosities are shown in Table S1. After processing at steady-state conditions, the molecular weight decreased with higher rotor speeds and decreasing orifice openings. These results followed the same trend as the melt flow index results. The unprocessed PLA had a molecular weight of 198,632 g/mol. With rotor speeds of 400 rpm to 800 rpm, the molecular weight decreased after processing by 11% to 20% for 15/7 HD rotors, by 6% to 11% for the 15/15 std rotors, and by 1% to 5% for the 15/7 std rotors. The reduction in molecular weight with increasing screw speed has been reported by many research groups when using twin-screw extruders [5,17,20,23]. The reduction in molecular weight was attributed to the effect of shearing on polymer melt caused a thermomechanical degradation. Conversely, Aldhafeeri et al. [25] reported an increase in molecular weight when increasing screw speed from 400 to 1000 rpm due to the longer residence time at lower screw speeds when using twin- and quad-screw extruders.

The molecular weight was also affected by orifice position (Figure 10b), with smaller openings producing greater reductions in molecular weight. For instance, when using the 15/7 HD rotors, the molecular weight decreased by 8% to 16% when the orifice opening decreased from 50% to 10%, respectively. The 15/15 std exhibited weight reductions of 6% to 14% for the same decrease in orifice opening. In contrast, the 15/7 std rotor design produced minimal molecular weight reduction of 3% to 8%—i.e., it showed minimal thermomechanical degradation and the highest retention of molecular weight. Smaller orifice openings allow greater backflow. Based on these results, the 15/7 HD and 15/15 std rotor designs were far more aggressive, as they produced more significant reductions in molecular weight.

**Figure 11:**
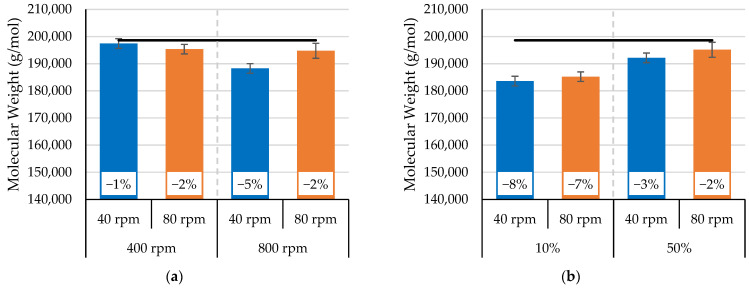
Molecular weight for the processed PLA as a function of extruder screw speed at different (**a**) 15/7 std rotor speeds and a fixed orifice position of 30% and (**b**) orifice positions with a constant 15/7 std rotor speed of 600 rpm.

Figure 11 shows the effect of extruder screw speed on the molecular weight of the processed PLA; the zero-shear viscosities are shown in Table S2. The results were similar to the melt flow index results reported for increasing the screw speed from 40 rpm to 80 rpm at a fixed orifice of 30% and increasing orifice positions at a constant rotor speed of 600 rpm. With the lower rotor speed of 400 rpm in the mixing section, increasing the screw speed decreased the molecular weight of the processed PLA by 1%. When the rotor speed in the mixer section was 800 rpm, however, increasing the screw speed helped marginally to minimize the reduction in molecular weight. The molecular weight retention increased from 5% to 2% when the screw speed increased from 40 to 80 rpm. In addition, with larger orifice openings (i.e., 50%), the molecular weight was higher compared to the molecular weight for the smaller opening of 10%; the greater retention of molecular weight was attributed to less flow restriction at the orifice. This reduction in flow restriction minimized backflow inside the mixer section and resulted in lower viscous dissipation. Overall, these results suggest that the extruder screw speed had an insignificant impact on the process-induced degradation of PLA. Based on the molecular weight and MFI results (Figure 9 and Figure 11), further study could help to understand the interaction of the mixing section and extruder section in the Farrel continuous mixer on the molecular weight retention of PLA.

The feeding of molten polymer (compared to unmelted pellets) to the single-screw extruder significantly impacted the effect of screw speed on the molecular weight of PLA. Velghe et al. [43] reported that, with a pellet-fed single-screw extruder, increasing screw speed from 20 to 40 rpm helped to minimize the reduction in molecular weight by 10%. This behavior was attributed to the fact that the higher screw speed helped to pump the polymer melt faster, resulting in a lower residence time and minimizing the reduction in molecular weight.

**Figure 12:**
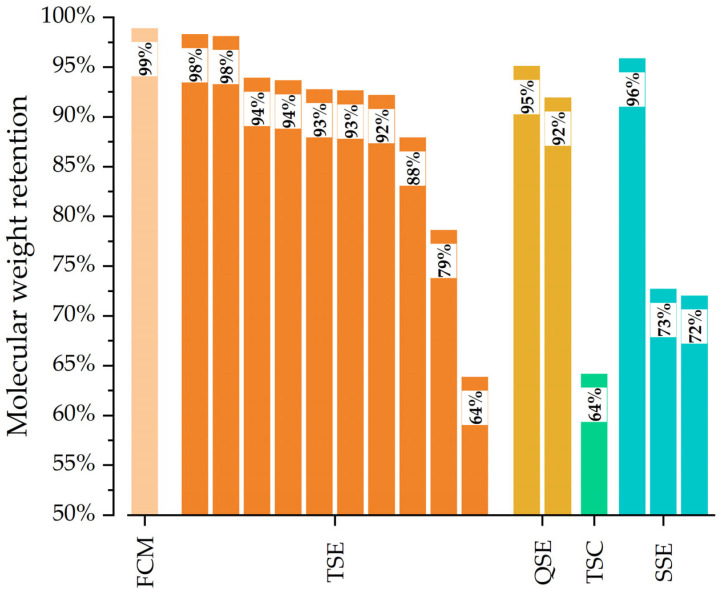
Molecular weight retention of processed PLA using Farrel continuous mixer (FCM), co-rotating twin-screw extruder (TSE) [1,5,21,25,30,44,45,46,47], quad-screw extruder (QSE) [25], counter-rotating twin-screw extruder (TSC) [30], and single-screw extruders (SSE) [30,43,48].

Figure 12 compares the molecular weight retention for PLA processed in the Farrel continuous mixer (FCM) with published results for PLA processed in other melt mixing systems. The melt mixing equipment includes co-rotating twin-screw extruders (TSEs), co-rotating quad-screw extruders (QSEs), counter-rotating twin-screw extruders (TSCs), and single-screw extruders (SSEs). With the 15/7 std rotor design and optimal rotor speed, extruder speed, and orifice opening, the Farrel continuous mixer exhibited 99% retention of PLA’s molecular weight. In contrast, the TSEs showed molecular weight retentions that ranged from 98% to 64%. This molecular weight retention varied with the extruder size and the screw programs. When using the same PLA grade (Ingeo™ Biopolymer, 2003D), screw diameter (35 mm), and feed rate as the FCM, TSE exhibited a 92% retention of molecular weight [44]. Larger reductions in molecular weight were probably due to screw programs with more kneading blocks (although the screw programs were not provided). With a quad-screw extruder, Aldhafeeri et al. [25] reported molecular weight retentions of 92% and 95% for feed rates of 2 kg/h and 4 kg/h, respectively. The TSC produced a low molecular weight retention of approximately 64% due to the high shear stresses associated with the “conveying elements” of the screw—which led to more significant polymer degradation. For the SSEs, molecular weight retention ranged from 72% to 96%. Since the diameters and L/Ds were 19 mm to 30 mm and 25:1 and 34:1, respectively, retention was most likely due to the evaluated PLA grades. Overall, the results demonstrate that the FCM exhibited higher molecular weight retention compared to all other mixing equipment.

### 3.3. FTIR Spectroscopic Analysis

As the PLA melt was processed using different processing conditions and rotor designs, the resultant rheological properties clearly exhibited molecular weight degradation. To analyze how these processing conditions affected PLA’s chemical structure, ATR-FTIR was used. FTIR spectra can indicate PLA chemical structure changes due to degradation. The specific FTIR spectral bands associated with degradation of PLA include the 1750 cm^−1^ band (C=O stretching of carbonyl groups), the 1080–1180 cm^−1^ band (C-O stretching of ester groups), and the 3100–3750 cm^−1^ band (O-H groups). The as-measured spectra are presented in the Supplemental Data (Figure 1, Figure 2, Figure 3, Figure 4 and Figure 5). These FTIR spectra were normalized by dividing the entire spectrum by the reference peak intensity. The reference peak was 1455 cm^−1^, which is assigned to the asymmetric bending of the methyl group (CH_3_); it was chosen because it remained stable after processing [22,39,40,41].

**Figure 13:**
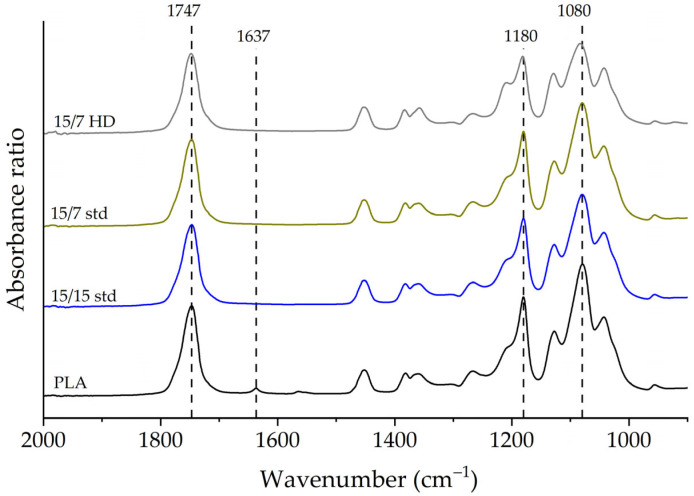
Fourier-transform infrared spectroscopy (FTIR) spectra for the processed PLA as a function of rotor design at a rotor speed of 800 rpm and orifice position of 30%.

After processing PLA, the peak at 3100–3750 cm^−1^ (O-H groups) remained unchanged, indicating no degradation due to hydrolysis. Since higher rotor speeds caused a significant reduction in molecular weight, Figure 13 displays the FTIR spectra for the PLA processed using different rotor designs at a rotor speed of 800 rpm. The most noticeable changes were at 1080, 1180, and 1750 cm^−1^. These changes in the intensities were associated with thermomechanical degradation resulting in random PLA chain scission when varying rotor speed, orifice position, and rotor designs [20,22,39,49]. In addition, with the 15/7 HD rotor design, a new peak was observed at 1250 cm^−1^; it corresponded to the C–O stretching vibration of the ester group. Moreover, after processing, the peak at 1630 cm^−1^ (corresponding to H-O-H bending vibrations) was not visible in the infrared spectra. The absence of this peak could indicate the occurrence of thermal chain scission at the C-O bond [46,50] or the presence of water molecules in unprocessed PLA [51].

**Figure 14:**
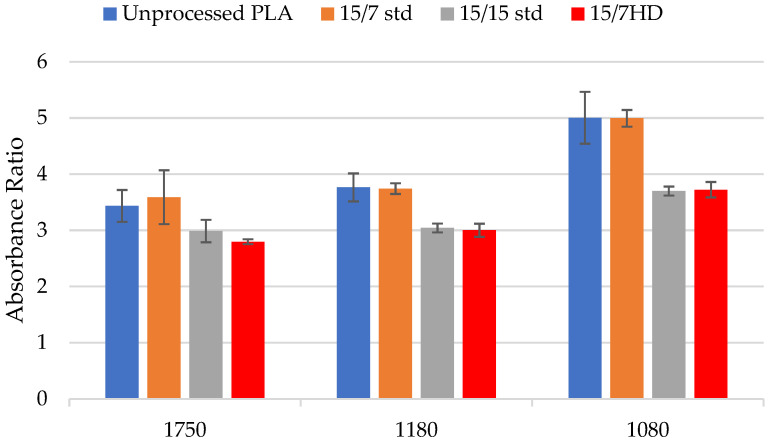
Absorbance ratio for the peaks at 1080, 1180, and 1750 cm^−1^ for unprocessed PLA and PLA processed using different rotor designs at a rotor speed of 800 rpm and orifice position of 30%.

The absorbance ratios in Figure 14 for peaks at 1080, 1180, and 1750 cm^−1^ provide information on how peak intensity varies with rotor design. These data showed significant differences in the chemical structure. Across the three rotor designs, the 15/15 std and 15/7 HD rotors produced significant changes for peaks at 1080, 1180, and 1750 cm^−1^. The absorbance ratios decreased significantly compared to those for unprocessed PLA. For the 15/7 std rotors, however, the absorbance ratios were comparable for the three peaks. This behavior was expected, as the 15/7 std rotors produced the lowest reduction in molecular weight.

### 3.4. Thermal Properties Analysis

Process-induced degradation can influence the thermal properties of the PLA since the thermal properties depend on the molecular weight [14]. Therefore, differential scanning calorimetry was used to identify possible changes in the thermal behavior of the processed PLA; typical heat flow-versus-temperature curves are shown in the Supplemental Materials (Figure S1: DSC curves for unprocessed PLA and processed PLA at 800 rpm and 10% orifice position using the 15/7 HD rotor). Table 4 and Table 5 present the melting enthalpy (ΔH_m_), the cold crystallization enthalpy (ΔH_cc_), cold crystallization temperature (T_cc_), melt temperatures (T_m_), and crystallinity values (X_c_) as a function of rotor speed and orifice position using different rotor designs. The unprocessed PLA did not exhibit a cold crystallization peak during the first heating, but it had a melting peak with a melting temperature of 152.3 °C. This behavior could have been due to the production process—i.e., the pellets had crystallized [25]. During the second heating cycle, however, no melting and cold crystallization peaks were present, possibly because the cooling rate of 10 °C/min did not allow the PLA chains to reorganize into crystalline regions because PLA has a low crystallization rate. For all processing conditions, the melt temperatures ranged from 150.3 °C to 151.7 °C, and the cold crystallization temperatures ranged from 121.5 to 123.9 °C. These results confirmed that these critical temperatures were not affected by processing PLA at various rotor speeds and orifice positions. Similar findings previously have been reported in the literature [25,52,53,54,55,56]. Changes in these temperatures are attributed to the PLA chains being broken into smaller fragments due to random chain scission reactions caused by thermomechanical degradation. These reactions lead to the formation of monomers, oligomers, and short chains, which can join the main chain to form a more spherical molecule due to the attached side chains [5]. Therefore, this mechanism did not affect the critical temperatures for the PLA processed in the FCM.

For the unprocessed PLA, the crystallinity was 39%. The same behavior was reported by Aldhafeeri et al. [25] and Sholaeiarani [7] when they characterized the same grade of PLA used in this work (grade: 2003D). Upon processing, the processed PLAs exhibited cold crystallization peaks, resulting in crystallinity ranging from 0.02% to 2.4%. The crystallinity marginally increased from 0.23% to 1.26% for the 15/15 std rotor, 0.04% to 0.22% for the 15/7 std rotor, and 0.29% to 1.3% for the 15/7 HD rotor samples with orifice openings of 50, 30, and 10%, respectively. The increase in crystallinity and the formation of cold crystallization peaks were most likely due to the greater molecular mobility caused by the processing variables and rotor designs, allowing the PLA chains to rearrange and form crystalline regions during the heating cycle. This behavior was reported when PLA was processed using a co-rotating twin-screw extruder [5,25]. Additionally, increasing the rotor speed increased crystallinity.

## 4. Conclusions

This work demonstrated the impact of mixing parameters (rotor speed, orifice position, and rotor design) when processing a biopolymer (PLA) using a Farrel continuous mixer. In addition, the effect of the screw speed for the single-screw extruder section in Farrel continuous mixer was investigated. Varying the rotor speed and orifice position caused increases in the melt flow index and reductions in the molecular weight of the processed PLA. The influence of extruder speed showed a minimal impact on the melt flow indices and molecular weight. Rotor design also contributed to the degradation of PLA. Among the three evaluated rotor designs, the 15/7 HD rotors had the highest impact on the molecular weight (i.e., a reduction of 14%), whereas the 15/7 std rotors reduced the molecular weight by only 5%. As confirmed by FTIR measurements, the dominant degradation pathway was chain scission, and greater chain scission was more pronounced with aggressive processing conditions and when using the 15/7 HD and 15/15 std rotors. The reductions in molecular weight, however, did not significantly affect the thermal properties of the processed PLA.

## Figures and Tables

**Table 1:** Overview about the experiments of the effect of mixer section parameters.

Trial	Rotor Design	Rotor Speed (rpm)	Orifice Opening (%)	Screw Speed (rpm)
1	15/15 std	400	30	40
2	15/15 std	600	30	40
3	15/15 std	800	30	40
4	15/15 std	600	10	40
5	15/15 std	600	50	40
6	15/7 std	400	30	40
7	15/7 std	600	30	40
8	15/7 std	800	30	40
9	15/7 std	600	10	40
10	15/7 std	600	50	40
11	15/7 HD	400	30	40
12	15/7 HD	600	30	40
13	15/7 HD	800	30	40
14	15/7 HD	600	10	40
15	15/7 HD	600	50	40

**Table 2:** Set temperatures for the continuous mixer trials.

	Std Rotors	HD Rotor
Mixer Chamber 1 (°C)	80	120
Mixer Chamber 2 (°C)	180	180
Orifice (°C)	180	180
Extruder Cylinder 1 (°C)	180	180
Extruder Cylinder 2 (°C)	180	180
Die (°C)	180	180

**Table 3:** Overview about the experiments of the effect of extruder section.

Trial	Rotor Design	Screw Speed (rpm)	Orifice Opening (%)	Rotor Speed (rpm)
1	15/7 std	40	10	600
2	15/7 std	80	10	600
3	15/7 std	40	50	600
4	15/7 std	80	50	600
5	15/7 std	40	30	400
6	15/7 std	80	30	400
7	15/7 std	40	30	800
8	15/7 std	80	30	800

**Table 4:** Thermal properties for the processed PLA as a function of rotor speed and rotor design.

Rotor Design	Rotor Speed (rpm)	ΔH_cc_ (J/g)	ΔH_m_ (J/g)	T_cc_ (°C)	T_m_ (°C)	X_c_ (%)
Unprocessed PLA (1st heating cycle)		-	36.3	-	152.3	39.03
Unprocessed PLA (2nd heating cycle)		-	-	-	-	-
15/15 std	400	16.7 ± 0.9	17.2 ± 0.6	123.6	150.8	0.58 ± 0.2
15/15 std	600	19.0 ± 0.4	19.7 ± 1	122.8	150.5	0.71 ± 0.3
15/15 std	800	16.3 ± 0.56	18.5 ± 0.7	122.5	151.1	2.41 ± 1
15/7 std	400	14.0 ± 0.8	14.0 ± 0.5	122.1	151.6	0.02 ± 0.01
15/7 std	600	18.5 ± 0.6	18.7 ± 0.7	121.7	150.3	0.18 ± 0.1
15/7 std	800	15.1 ± 0.9	16.3 ± 0.23	122.1	151.5	1.28 ± 0.6
15/7 HD	400	14.2 ± 0.6	15.1 ± 0.4	123.2	151.0	0.97 ± 0.5
15/7 HD	600	12.5 ± 0.8	13.5 ± 0.4	122.9	151.6	1.08 ± 0.5
15/7 HD	800	16.8 ± 0.9	17.8 ± 0.9	121.5	150.6	1.10 ± 0.6

**Table 5:** Thermal properties for the processed PLA as a function of orifice position and rotor design.

Rotor Design	Orifice Opening (%)	dH_cc_ (J/g)	dH_m_ (J/g)	T_cc_ (°C)	T_m_ (°C)	X_c_ (%)
15/15 std	10	18.2 ± 0.8	19.4 ± 0.5	121.7	150.6	1.26 ± 0.6
15/15 std	30	19.0 ± 0.4	19.7 ± 0.8	122.8	150.5	0.71 ± 0.3
15/15 std	50	16.8 ± 0.7	17.0 ± 1	123.9	151.7	0.23 ± 0.2
15/7 std	10	18.6 ± 0.4	18.8 ± 0.5	122.2	150.9	0.22 ± 0.1
15/7 std	30	18.5 ± 0.7	18.7 ± 0.5	121.7	150.3	0.18 ± 0.09
15/7 std	50	17.6 ± 0.5	17.7 ± 0.7	123.5	151.1	0.04 ± 0.02
15/7 HD	10	18.2 ± 0.6	19.4 ± 0.2	122.3	150.5	1.31 ± 0.6
15/7 HD	30	12.5 ± 0.5	13.5 ± 0.4	123.9	151.6	1.08 ± 0.3
15/7 HD	50	16.7 ± 0.8	16.9 ± 0.4	123.8	150.7	0.29 ± 0.1

## Data Availability

Data are contained within the article and Supplemental Data.

## Supplementary Materials

The following supporting information can be downloaded at: https://www.mdpi.com/article/10.3390/polym17111568/s1, Figure S1: DSC curves for unprocessed PLA and processed PLA at 800 rpm and 10% orifice position using the 15/7 HD rotor; Table S1: Zero shear viscosity as a function of rotor speed and rotor design; and Table S2: Zero shear viscosity as a function of orifice position and rotor design.
