# Supplementary material for: Effect of Continuous Mixer Design and Parameters on the Degradation of Polylactic Acid
Source: Polymers (Basel). 2025 Jun 4;17(11):1568. doi: 10.3390/polym17111568 (PMC12158169; doi:10.3390/polym17111568)
Supplement: Supplementary file 1 [file polymers-17-01568-s001.zip › polymers-3657063-supplementary.pdf]

Effect of Continuous Mixer Design and Mixing Parameters on Degradation of Polylactic Acid

Mansour Alotaibi [1], Jainam Shah [1], Aniket Sadani [1], and Carol Forance Barry [1,*]

[1] Department of Plastics Engineering, University of Massachusetts Lowell, Lowell, MA 01854, USA
* Correspondence: Carol_Barry@uml.edu

Table S1: Zero-shear viscosity as a function of rotor speed and rotor design

Rotor Speed (rpm)	Rotor Design	Zero-shear Viscosity (Pa.s)
400	15/15 std	4580.5
600	15/15 std	4325.1
800	15/15 std	3866.9
400	15/7 std	5557.2
600	15/7 std	4831.7
800	15/7 std	4726.4
400	15/7 HD	3882.0
600	15/7 HD	3421.7
800	15/7 HD	2685.5

Table S2: Zero-shear viscosity as a function of orifice position and rotor design

Orifice Position (%)	Rotor Design	Zero-shear Viscosity (Pa.s)
10	15/15 std	3333.4
30	15/15 std	4325.1
50	15/15 std	4570.2
10	15/7 std	4339.4
30	15/7 std	4831.7
50	15/7 std	5068.9
10	15/7 HD	3185.0
30	15/7 HD	3421.8
50	15/7 HD	4250.5

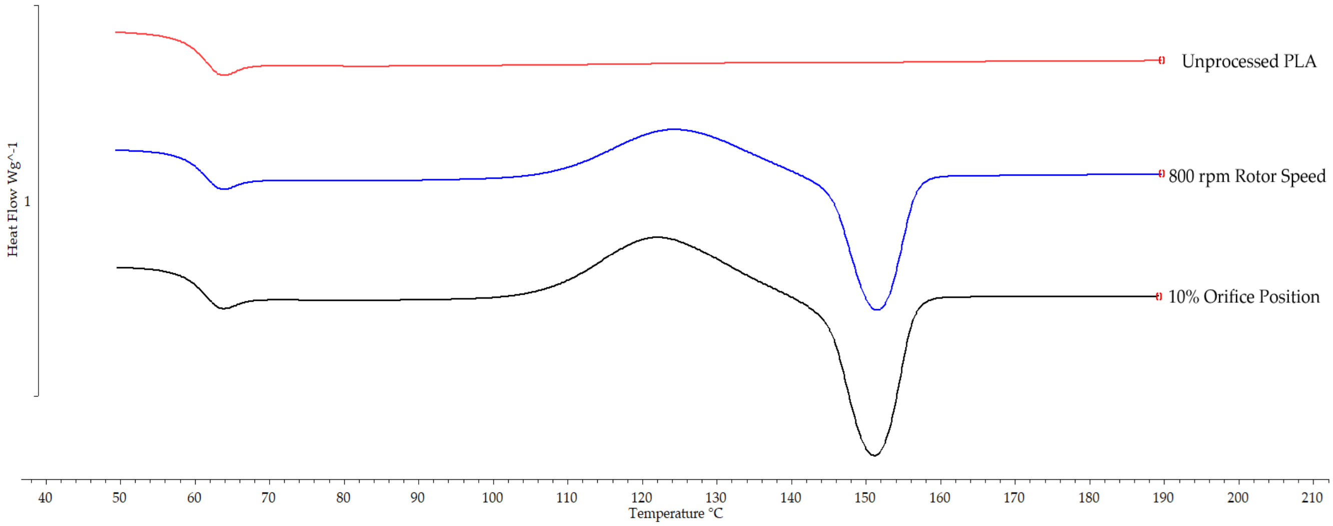

Figure S1: DSC curves for unprocessed PLA and processed PLA at 800 rpm and 10% orifice position using the 15/7 HD rotor.
